# Wire-in-Wire TiO_2_/C Nanofibers Free-Standing Anodes for Li-Ion and K-Ion Batteries with Long Cycling Stability and High Capacity

**DOI:** 10.1007/s40820-021-00632-4

**Published:** 2021-04-09

**Authors:** Die Su, Yi Pei, Li Liu, Zhixiao Liu, Junfang Liu, Min Yang, Jiaxing Wen, Jing Dai, Huiqiu Deng, Guozhong Cao

**Affiliations:** 1 National Base for International Science and Technology Cooperation, National Local Joint Engineering Laboratory for Key Materials of New Energy Storage Battery, Hunan Province Key Laboratory of Electrochemical Energy Storage and Conversion, School of Chemistry, Xiangtan University, Xiangtan, 411105 People’s Republic of China; 2 Department of Materials Science and Engineering, University of Washington, Seattle, WA 98195-2120 USA; 3 College of Materials Science and Engineering, Hunan University, Changsha, 410082 People’s Republic of China; 4 School of Physics and Electronics, Hunan University, Changsha, 410082 People’s Republic of China

**Keywords:** Free-standing TiO_2_/C nanofiber, Li-ion battery, K-ion battery, First-principles calculation, Full cells

## Abstract

Wearable and portable mobile phones play a critical role in the market, and one of the key technologies is the flexible electrode with high specific capacity and excellent mechanical flexibility. Herein, a wire-in-wire TiO_2_/C nanofibers (TiO_2_ ww/CN) film is synthesized via electrospinning with selenium as a structural inducer. The interconnected carbon network and unique wire-in-wire nanostructure cannot only improve electronic conductivity and induce effective charge transports, but also bring a superior mechanic flexibility. Ultimately, TiO_2_ ww/CN film shows outstanding electrochemical performance as free-standing electrodes in Li/K ion batteries. It shows a discharge capacity as high as 303 mAh g^−1^ at 5 A g^−1^ after 6000 cycles in Li half-cells, and the unique structure is well-reserved after long-term cycling. Moreover, even TiO_2_ has a large diffusion barrier of K^+^, TiO_2_ ww/CN film demonstrates excellent performance (259 mAh g^−1^ at 0.05 A g^−1^ after 1000 cycles) in K half-cells owing to extraordinary pseudocapacitive contribution. The Li/K full cells consisted of TiO_2_ ww/CN film anode and LiFePO_4_/Perylene-3,4,9,10-tetracarboxylic dianhydride cathode possess outstanding cycling stability and demonstrate practical application from lighting at least 19 LEDs. It is, therefore, expected that this material will find broad applications in portable and wearable Li/K-ion batteries.

**Supplementary Information:**

The online version contains supplementary material available at 10.1007/s40820-021-00632-4.

## Highlights

- The unique wire-in-wire structure endows TiO_2_/C nanofibers film with superior mechanical flexibility.
- The wire-in-wire TiO_2_/C nanofibers (TiO_2_ ww/CN) film shows outstanding electrochemical performances as free-standing anodes for Li/K-ion batteries and full cells.
- The TiO_2_ ww/CN film shows an extremely high pseudocapacitance contribution ratio in K-ion batteries.

**Supplementary Information:**

The online version contains supplementary material available at 10.1007/s40820-021-00632-4.

## Graphical abstract

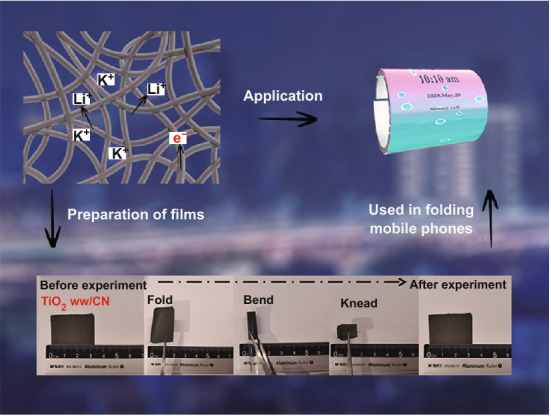

## Introduction

With the development of flexible electronic devices or wearable devices, the studies for flexible energy-storage devices are becoming increasingly attractive [1, 2]. As one of the most efficient energy storage devices, flexible Li-ion batteries (LIBs) gradually become all-important in the field of energy storage. Recently, K-ion batteries (KIBs) have attracted masses of attention because of their abundant raw materials in natural resources, similar working mechanisms, and approximate standard reduction potential (lithium: − 3.04 V vs. E^0^, potassium: − 2.93 V vs. E^0^) compared with LIBs [3–5]. The flexible electrodes with high-performance are essential for the applications of flexible advanced secondary batteries.

Binding-free and free-standing anode materials for LIBs, including layered transition metal oxides and sulfides [6, 7], have made great breakthroughs in flexible and wearable devices and other small portable electronic products. However, binding-free and free-standing anode materials for KIBs principally focus on red phosphorus or black phosphorus-doped carbon [8, 9], the reports about oxides or sulfides as free-standing anode materials are few. Titanium dioxide (TiO_2_) has distinct advantages in battery materials because of its wonderful natural abundance, low cost, and stable crystal structure. Thus, it has been extensively reported as anode electrode materials for LIBs and commonly exhibited long cycling stability due to small changes in volume size during repeated insertion/extraction of the crystal structure [10–13]. Nevertheless, theoretical specific capacity of TiO_2_ is 336 mAh g^−1^ based on inserting 1 mol Li per molecule, its actual specific capacity is usually not satisfied and the Li insertion amount is frequently limited below 0.5 mol [14]. Although TiO_2_ layer on N-doped carbon foams [15], EOG/TiO_2_(B) nanosheets [16], and N-doped TiO_2_/rGO [17] have been reported as free-standing anodes for LIBs, their specific capacity and flexibility have a lot of room for improvement.

Making every effort to investigate, there are only three reports about TiO_2_ as anodes for KIBs. Hierarchical TiO_2_-C micro-tubes [18], lepidocrocite-type layered TiO_2_ [19], and MXene-derived TiO_2_/RGO [20] have been synthesized and studied as anode materials for KIBs. It is noteworthy that free-standing TiO_2_ anode in K-ions battery has not been reported as we know. This is a great challenge to achieve high mechanical flexibility between TiO_2_ nanoparticles and carbon matrix materials. Herein, using selenium as a structural inducer, wire-in-wire TiO_2_/C nanofibers (denoted as TiO_2_ ww/CN) film has been synthesized via electrospinning. Without a traditional coating process, it possesses the characteristics of long cycle life and high capacity as independent electrode anodes for Li/K half cells and full cells. Excellent lithium and potassium storage performance and strong mechanical flexibility make it become a potential flexible anode candidate.

## Experimental Section

### Synthetic Section

All the reagents, including tetra-n-butyl titanate (TBOT, C_16_H_36_O_4_Ti, Kermel), *N*,*N*-dimethylformamide (DMF, C_3_H_7_NO, Sinopharm, China), polyvinylpyrrolidone (PVP, (C_6_H_9_NO)_*n*_, MW = 1,300,000, Alfa Aesar), commercial LiFePO_4_ (LFP, BTR New Material Group Co., Ltd, China), perylene-3,4,9,10-tetracarboxylic dianhydride (PTCDA, Aladdin, China), glacial acetic acid (HAC, CH_3_COOH, Kermel), and selenium powder (Se, Macklin, 200 mesh) are analytically pure grade and used directly.

**Scheme 1:**
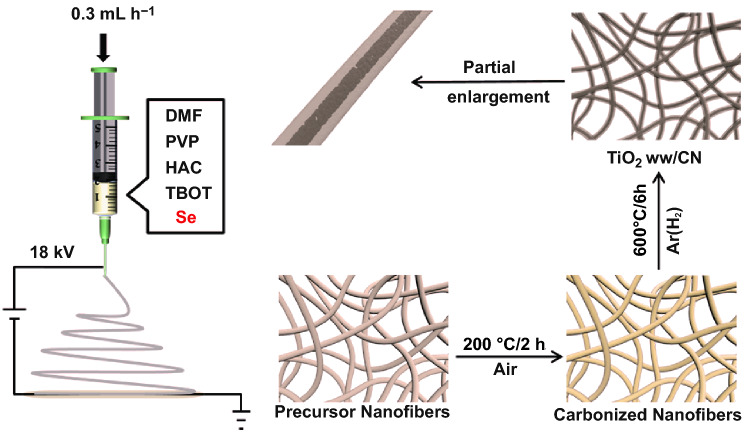
Schematics illustration of the process used to produce the TiO_2_ ww/CN film

The synthetic route is shown in Scheme 1. First, 0.8 g PVP was dissolved in 9 mL DMF via stirring to obtain a clear transparent solution. Then, 1.5 mL CH_3_COOH and 2 mL TBOT were added to the solution slowly. After 10 min, 0.48 g selenium powder was added to the above solution with stirring at 60 °C for 24 h and ultrasonic dispersion for 1 h. The digital photo of the precursor solution (including DMF, PVP, TBOT, HAc, and Se) shown in Fig. S1a confirmed the complete dissolution of Se powder in the colloids of DMF and PVP, while the compared one without PVP showed considerable undissolved Se powder in Fig. S1b. The precursor solution was spun under 18 kV to obtain the precursor nanofibers film. Then, the precursor nanofiber film was stabilized in air at 200 °C/2 h. In the end, the TiO_2_ ww/CN film was obtained by annealing the stabilized nanofibers film in Ar (H_2_) (95:5) atmosphere at 600 °C/6 h. The thickness of the TiO_2_ ww/CN film is about 0.083 mm (as shown in Fig. S1c). For comparative study, common TiO_2_/C nanofibers (TiO_2_/CN) film was obtained from the precursor solution without Se adding by the above procedure. Besides, the PTCDA used as cathode materials in K full cell (KFC) was pretreated by annealing at 450 °C for 4 h in Ar [5].

### Structure and Morphology Characterization

The crystal structure of all samples was characterized by X-ray diffraction (XRD, Rigaku, Ultima IV with D/teX Ultra with Cu-Kα radiation) at a scan rate of 5° min^−1^. The Raman spectra of the sample were characterized by the microlaser Raman spectrometer (Raman, Renishaw inVia, plc England). The elemental distribution on the surface of the nanofibers was characterized by X-ray photoelectron spectroscopy (XPS, Kratos Axis Ultra DLD, Japan) (hν = 1486.6) using a Kratos Axis Ultra DLD spectrometer of a monochromatic Al Kα X-ray source. The content of the carbon and selenium was measured by an elemental analyzer (TG, NETZSCH STA 409 PC/PG, Germany). The morphology and microscopic structure of the product were presented by field emission scanning electron microscopy (FE-SEM, Hitachi, SU8010, Japan) and transmission electron microscopy (FE-TEM, FEI-Tecnai G2 TF20, America). The corresponding element mapping was performed by the high-angle annular dark-field (HAADF) and selected area electron diffraction (SAED). The specific surface area and the porous structure were calculated by a specific surface and aperture analyzer (BET, Quadrasorb SI-3MP, USA). And the pore size distribution was estimated with BJH method.

### Electrochemical Tests

Without using any binder, conductive additive, and metal current collector, the obtained samples were punched to discs with a diameter of 10 mm and directly applied as working electrodes. And the area loading of active materials in the electrodes is about 0.7 mg cm^−2^. The slurry is composed of the 70 wt% of the active substance (LFP or PTCDA), 20 wt% of carbon black, 10 wt% of polyvinylidene fluoride, and appropriate *N*-methyl-2-pyrrolidone is pasted on aluminum foil to form cathodes for Li/K full cells. The mass loading of active materials for LFP electrode is about 0.77 mg cm^−2^ and PTCDA electrode is about 1.4 mg cm^−2^. The Li half cell (LHC) was fabricated by sandwiching a separator (Whatman GF/A) between lithium metal and working electrode into CR2025 coin cell, and the 1.0 M LiPF_6_ with ethylene carbonate/dimethyl carbonate (EC/DMC) (1:1 vol %) was employed as the electrolyte. The lithium metal was replaced by the LFP cathode to assemble Li full cell (LFC). The CR2032 K half cell (KHC) was assembled using a working electrode, potassium metal, glass fiber membrane (Whatman GF/D) as a separator, and 1.0 M KFSI in ethylene carbonate/dimethyl carbonate (EC/DMC) (1:1 vol%) as an electrolyte. The potassium metal was replaced by the PTCDA cathode to fabricate KFC. To ensure the cell balance, setting the weight ratio of cathode to anode active material is about 2:1 in LFCs and 1.1:1 in KFCs. All cells were assembled in the glovebox that both moisture and oxygen concentration were less than 0.1 ppm. The Neware BT3008W battery test system (China) was measured to test the constant current discharge/charge process. The Chenhua CHI660E Analyzer (China) was performed on cyclic voltammetry (CV) and A.C. impedance (frequency range: 0.01 Hz to 100 kHz; amplitude: 5 mV).

### Computational Details

All first-principles simulations (DFTs) are performed by the Vienna Ab-initio Simulation Package [21, 22]. The projector augmented-wave (PAW) method [23] was used to demonstrate the ion–electron interactions. The Perdew−Burke−Ernzerhof (PBE) functional [24] was employed for describing the electron–electron exchange correlations. The onsite Coulomb interaction is considered in the present study to describe the localized *d* electrons of transition metals, and the *U*_eff_ parameters were set to 2 eV. In all DFT simulation, making the cutoff energy of the plane wave basis sets equals 400 eV, and the density of k points equals to 0.15 Å^−1^. The residual force for optimizing atom positions was less than 0.02 eV Å^−1^.

## Results and Discussion

### Sample Characterization

**Fig. 1:**
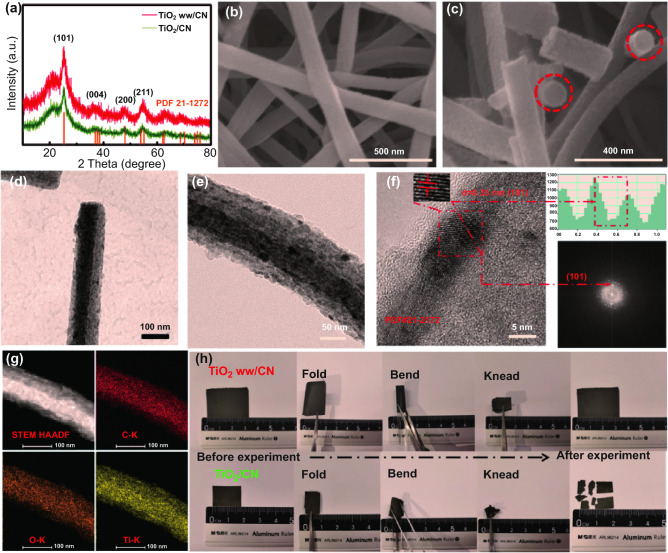
**a** XRD patterns of TiO_2_ ww/CN and TiO_2_/CN; FE-SEM images of **b** TiO_2_ ww/CN film and **c** the fracture cross section nanofibers for TiO_2_ ww/CN; **d**, **e** FE-TEM images, **f** HRTEM images, and **g** EDX elemental mapping images of C, O, Ti of TiO_2_ ww/CN; **h** the digital photos of TiO_2_ ww/CN film and TiO_2_/CN film before and after the experiment of completely folded, bent and kneaded in sequence

The XRD patterns show crystallographic information in Fig. 1a. The diffraction peaks of both TiO_2_/CN and TiO_2_ ww/CN are fitted well with the anatase (JCPDS No. 21-1272) without impure phases, indicating that pure-phase TiO_2_ is successfully prepared by electrospinning method. The FE-SEM image shows that the precursor nanofibers of the TiO_2_ ww/CN (Fig. S2a) have a large length-diameter ratio with diameters of about 200 nm. However, the diameters of the carbonized nanofibers (Fig. 1b) are significantly reduced to 160 nm. The TiO_2_/CN film and its precursor show similar morphologies with that of TiO_2_ ww/CN film and its precursor (Fig. S2b, c). To further investigate the morphology of TiO_2_ ww/CN film, the FE-SEM image of the fractured cross section of nanofibers is shown in Fig. 1c, indicating the nanofibers are solid. As shown in the TEM image (Fig. S3a), TiO_2_ nanoparticles in TiO_2_/CN have been uniformly embedded into the carbon nanofibers matrix. The TiO_2_ nanoparticles have diameters of ~ 6 nm and the lattice fringe of 3.5 Å from the HRTEM image (Fig. S3b) could be assigned to the (101) planes of anatase-phase TiO_2_. Meanwhile, the corresponded fast Fourier transform (FFT) pattern shows one set of polycrystalline rings that match well with the layer distance of (101) planes. The EDX element mapping image shown in Fig. S3c has indicated the even dispersion of C, Ti, and O within nanofiber, suggesting no element segregation in TiO_2_/CN. The TEM images of TiO_2_ ww/CN are shown in Fig. 1d, e. Despite a similar one-dimensional morphology (diameter of ~ 160 nm) of TiO_2_ ww/CN with that of TiO_2_/CN, the core of TiO_2_ ww/CN shows much higher contrast than that of the edge region, suggesting that TiO_2_ is probably aggregated in the core region and form a unique wire-in-wire nanostructure. The phase constitution in the interior region of TiO_2_ ww/CN is further investigated through HRTEM analysis (Fig. 1f), from which the lattice fringes with an interplanar spacing of 3.5 Å is consistent with the (101) plane of anatase TiO_2_, and the rings in FFT pattern are attributed to (101). The EDX element mapping images (Fig. 1g) confirm that the C, Ti, O elements are distributed in the whole nanofiber. To confirm the distribution of C and Ti elements, the TEM–EDX mapping line scan (Figure S4) is carried out. According to the scanning route in Fig. S4a, the EDX lines of the atomic ratio of the elements (C and Ti) were shown in Fig. S4b. It was observed that the C element increases gradually from inside to outside, while Ti element decreases gradually from inside to outside. This further supports the unique wire-in-wire nanostructure. To further investigate the inner structure of TiO_2_ ww/CN film, it was calcined in air at 400 °C for 2 h to remove the carbon matrix. The digital photo and TEM results of the residues are shown in Fig. S5. The black TiO_2_ ww/CN film turns into a white TiO_2_ film after burning in air (Fig. S5a), fortunately, the film structure is still intact. FE-TEM images in Fig. S5b, c demonstrate that the nanofibers (~ 60 nm) are composed of nanoparticles with diameters of about 6 nm. The interplanar spacing of HRTEM image (Fig. S5d) and SAED pattern (Fig. S5e) is all corresponding to (101) planes of anatase TiO_2_ phase (JCPDS No. 21-1272). EDX elemental mapping image shown in Fig. S5f indicates the nanowires are made up of Ti, O, and C elements. Figure 1h shows the digital photos of TiO_2_ ww/CN film and TiO_2_/CN film before and after the experiment of completely folded, bent, and kneaded in sequence. The TiO_2_/CN film is broken and couldn’t recover after the experiment, while the original state TiO_2_ ww/CN film is well-reserved, demonstrating the remarkable mechanical flexibility of TiO_2_ ww/CN film. More detailed information on the mechanical flexibility of TiO_2_/CN film and TiO_2_ ww/CN film can be seen in Video S1. It's worth noting that the TiO_2_ ww/CN film is intact after bending, folding, and kneading sequentially.

**Fig. 2:**
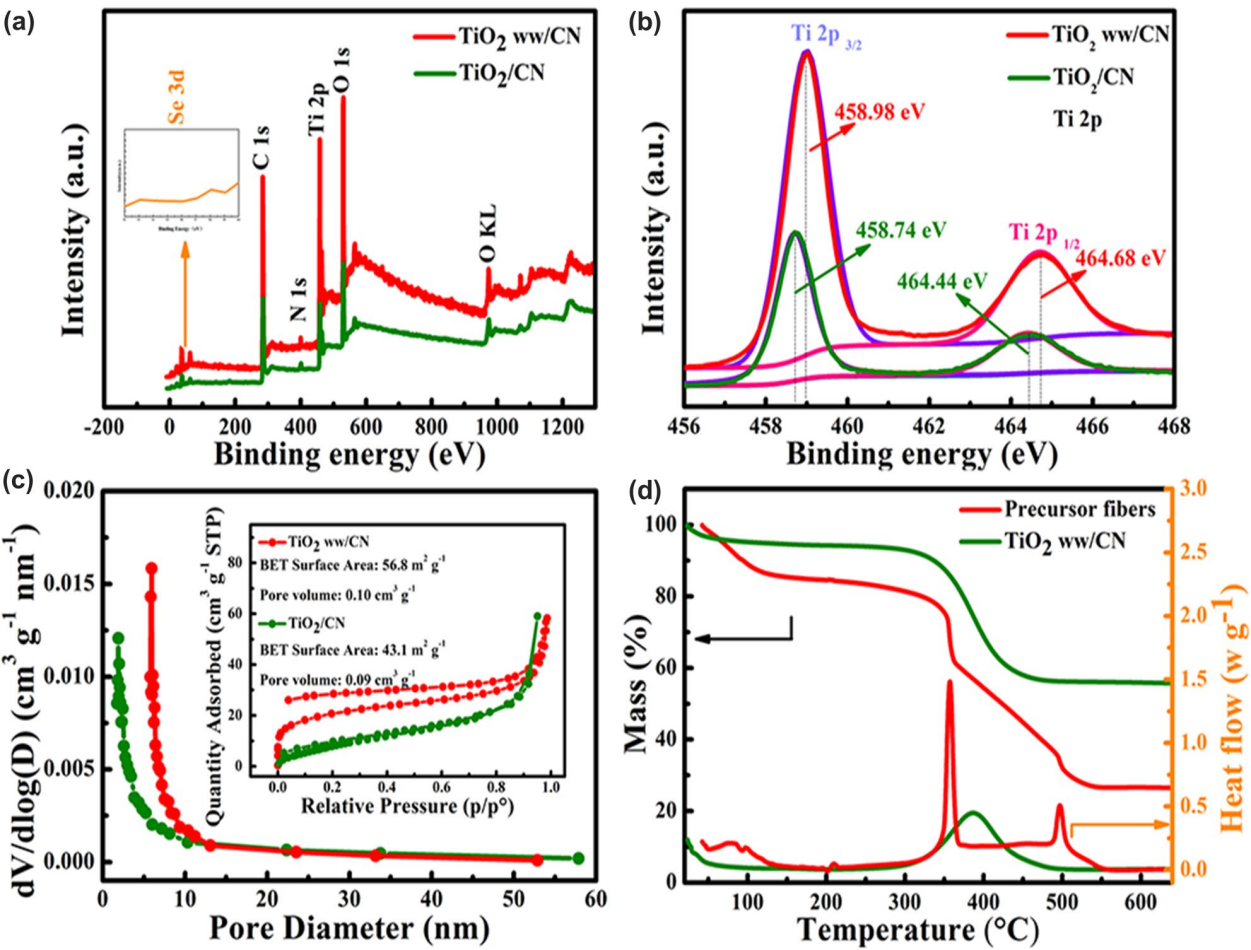
**a** XPS survey spectrums, **b** the high-resolution Ti 2*p* of TiO_2_ ww/CN and TiO_2_/CN; **c** the pore volume distribution of both TiO_2_ ww/CN and TiO_2_/CN, corresponding N_2_ adsorption/desorption curves (inserted image); **d** TG and DTA of TiO_2_ ww/CN and precursor fibers in air at a rate of 5 °C min^−1^

Therein, it can conclude that the addition of Se powder has induced the formation of specific wire-in-wire configuration in TiO_2_ ww/CN with superior mechanical performance. Even that the crucial role of Se powder could be confirmed by comparing the phase distribution of TiO_2_ ww/CN and TiO_2_/CN, the mechanism is still unclear. In consequence, further comparisons are designed and carried out on all the precursor, intermediate and as-synthesized products. The element composition and corresponding chemical states of TiO_2_ ww/CN and TiO_2_/CN were explored by XPS. The survey spectrums in Fig. 2a demonstrate that two samples have the same signal peaks of Ti, C, and O elements which are consistent with EDS from FE-TEM (Fig. S6). No signal of Se is detected in the spectra of TiO_2_ ww/CN, suggesting that Se is thoroughly evaporated. To further probe the electronic states of Ti and C, high-resolution C 1*s* and Ti 2*p* spectra are shown in Figs. S7 and 2b, respectively. Both TiO_2_/CN and TiO_2_ ww/CN demonstrate three conditions of C: C–C bond (284.80 eV), C–O bond (286.20 eV), and O–C=O bond (288.00 eV), and the main peak at 284.80 eV is attributed to unoxidized carbon, while the signal from 286.20 and 288.00 eV is traced from incomplete carbonization of PVP [25–28]. It can be observed in Fig. 2b that Ti 2*p* spectra of TiO_2_ ww/CN are slightly different from TiO_2_/CN. Two peaks locating in 458.74 and 464.44 eV for TiO_2_/CN correspond to the Ti 2*p*3/2 and Ti 2*p*1/2 peaks of TiO_2_, respectively. For TiO_2_ ww/CN, these two peaks shift to 458.98 and 464.68 eV, respectively. The higher binding energy of Ti 2*p* for TiO_2_ ww/CN is maybe concerned with the oxygen vacancies caused by the addition of structural inducers[29]. Consequently, XPS measurements are carried out on the precursor solution to probe the chemical state change of Se upon calcination (Fig. S8). The Se 3*d* spectra obtained from the precursor solution showed a characterization peak at 53.90 eV, which is corresponded to Se^0+^ in elementary Se [30, 31]. Therefore, interior nanofibers and exterior nanofibers of TiO_2_ ww/CN are comprised of TiO_2_ and carbon matrix. The formation of a unique hierarchical structure may be attributed to the selenium-gradient evaporation caused by annealing. As shown in Figs. S1 and S9 (FE-SEM image EDAX of the precursor nanofibers of TiO_2_ ww/CN), the selenium element is distributed uniformly in the precursor solution and precursor nanofibers after electrospinning. However, because of the low sublimation temperature of Se, the outer layer of the fibers may be enriched with selenium with the increase in temperature during annealing. Contrarily, titanium oxides would be concentrated on the inner layer of the fiber because of their high thermal-stability. Then, the embedded selenium in exterior gradually sublime with the temperature rises during annealing to form the unique wire-in-wire structure.

The N_2_ adsorption/desorption curves and corresponding pore volume distribution of both TiO_2_ ww/CN and TiO_2_/CN are shown in Fig. 2c to compare the surface condition. The specific surface area value of TiO_2_ ww/CN is 56.8 m^2^ g^−1^ with the pore volume value of 0.10 cm^3^ g^−1^, which is higher than that of TiO_2_/CN (43.1 m^2^ g^−1^, 0.09 cm^3^ g^−1^). This larger specific surface area and pore volume are probably traced from the formation of the void by the evaporation of selenium. Besides, it was observed from the pore volume distribution that TiO_2_/CN have micropores, while TiO_2_ ww/CN has mesopores and micropores. The TG–DTA curves of both TiO_2_ ww/CN and its precursor fibers in air are shown in Fig. 2d. The TG curve of precursor fibers shows two obvious stages of weight loss, corresponding to two heat flow peaks at 350 and 500 °C in the DTA curves, respectively. The weight losses at about 350 and 500 °C could be attributed to the decomposition of PVP and the sublimation of Se, respectively. Thus, selenium could be expected to be completely evaporated during the annealing process at 600 °C. The TG-DSC curves of TiO_2_ ww/CN and TiO_2_/CN in air are shown in Fig. S10. Both samples showed a similar weight loss process, within which the region before 200 °C is attributed from the loss of physically adsorbed water, and the weight loss between 300 and 500 °C with a heat flow signal peak at about 400 °C in the DSC curve caused by the burning of carbon in air. Thus, it can be calculated the carbon contents of those two samples are very close which are about 40.3% and 40.0%, respectively. The Raman spectra shown in Fig. S11 exhibited a similar intensity ratio of D band peak and G band peak (*I*_D_/*I*_G_) in TiO_2_ ww/CN and TiO_2_/CN (0.856), implying the comparable degree of ordering for carbon in TiO_2_ ww/CN and TiO_2_/CN.

### Electrochemical Evaluation in LHCs and Full Cells

**Fig. 3:**
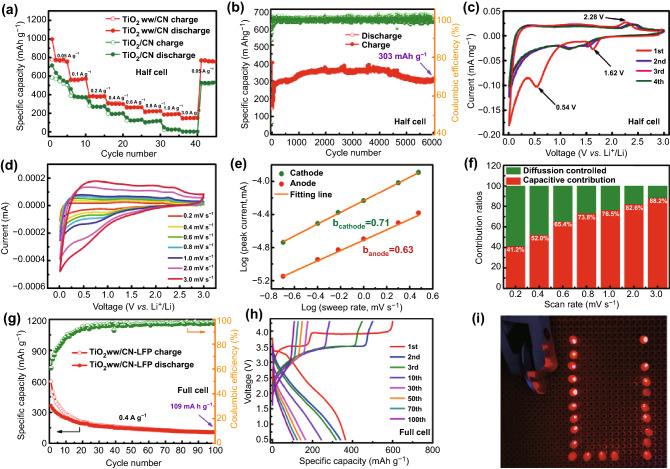
TiO_2_ ww/CN film and TiO_2_/CN film in LHCs: **a** rate performance at various current densities from 0.05 to 3 A g^−1^, at last returning back to 0.05 A g^−1^, **b** long cycling performance at 5 A g^−1^ for TiO_2_ ww/CN electrode; TiO_2_ ww/CN film in LHCs: **c** CV curves of TiO_2_ ww/CN film at 0.1 mV s^−1^, **d** CV curves of TiO_2_ ww/CN film from 0.2 to 3.0 mV s^−1^, **e** the log-linear relation in sweep rate ~ peak current, and **f** columnar diagram of contribution ratios from 0.2 to 3.0 mV s^−1^; (−)TiO_2_ ww/CN-LFP(+) full cell: **g** cycling performance, and **h** corresponding discharge–charge voltage profiles at 0.4 A g^−1^, **i** the digital picture of 22 LEDs after being lighted

Figure 3a compares the rate performance between the TiO_2_ ww/CN electrode and the TiO_2_/CN electrode. At 0.05, 0.1, 0.2, 0.4, 0.6, and 0.8 A g^−1^, the TiO_2_ ww/CN electrode can reach the capacities of 753, 574, 381, 300, 265, and 225 mAh g^−1^, respectively, which are much higher than those of TiO_2_/CN electrode. Even at 1, 3, and return to 0.05 A g^−1^, the TiO_2_ ww/CN electrode delivers the discharge specific capacities of 192, 149, and 750 mAh g^−1^, respectively. The cycling performance of the TiO_2_ ww/CN electrode and TiO_2_/CN electrode at 0.4 A g^−1^ is shown in Fig. S12a. The TiO_2_/CN electrode exhibits a charge capacity of 224 mAh g^−1^ with initial coulombic efficiency (ICE) of 44.8%, which are much lower than those of TiO_2_ ww/CN electrode. After the first cycle, the capacities of the TiO_2_ ww/CN electrode increase first and then keep stable gradually. It delivers a high discharge capacity of 497 mAh g^−1^ after 1000 cycles with coulombic efficiency (CE) of nearly 100%, demonstrating excellent cyclability. Figure 3b shows the long-term cycling (6000 cycles) of the TiO_2_ ww/CN electrode at 5 A g^−1^. The performance of the first 500 cycles is shown in Fig. S12b for better observation. It shows an initial discharge capacity of 401 mAh g^−1^ and an initial charge capacity of 183 mAh g^−1^. The discharge capacity at the second cycle is 116 mAh g^−1^, then the capacity significantly increases to 280 mAh g^−1^ at the 144th cycle. The increase may be due to the following reasons [32–36]: the activation of the electrolyte at the electrode, sectional reversible reactions of SEI, and the reversible interfacial Li-ion intercalation reactions. After that, the discharge capacity gradually increases to 367 mAh g^−1^ at nearly 4000 cycles. The TiO_2_ ww/CN electrode shows an outstanding cyclability and discharge capacity as high as 303 mAh g^−1^ after 6000 cycles. Figure S12c displays the discharge/charge curves of TiO_2_ ww/CN in different cycles at 0.4 A g^−1^. TiO_2_ ww/CN electrode shows an initial discharge capacity of 708 mAh g^−1^, then it shows a charge capacity of 383 mAh g^−1^ with ICE of 54%. Consistent with Fig. S12a, the large initial irreversible capacity loss is mainly in line with the solid electrolyte interphase (SEI). This may also be due to the larger specific surface area [37, 38] and the irreversible sites in Li^+^ ion storage [39]. The discharge/charge profiles at subsequent cycles are overlapping well, and the coulombic efficiencies reach nearly 100%. To explore the Li-storage properties, CV and A.C. impedance of the assembled LHCs were carried out. Figure 3c is the CV curves of TiO_2_ ww/CN. A pair of reduction/oxidation peaks at 1.62/2.28 V in the first scan can be ascribed to Li^+^ insertion/extraction in the anatase TiO_2_ [40–42]. There is a significant cathodic peak at about 0.54 V in the first cycle, which disappears in the following scans. It can be attributed to the generation of SEI film, which is formed from the irreversible reaction of electrode material and electrolyte [43–46]. After the first scan, the CV curves nearly overlapping suggesting that the TiO_2_ ww/CN electrode has good electrochemical reversibility. The lithium storage performance in this work exceeds the theoretical value, thus the pseudocapacitance behavior is analyzed in Fig. 3d–f and S12d to investigate the reason. CV curves at 0.2–3.0 mV s^−1^ are explored in Fig. 3d to research the kinetic performance of the TiO_2_ ww/CN electrode. In order to judge whether there is capacitive diffusion behavior in this process [11, 47, 48], the peak current (*i*) under the corresponding peak potential is taken to calculate from Eq. 1:

1 $$i = a\nu^{b}$$

where *i* is the peak current, *ν* is the scan rate, *a* and *b* are the constant. Therefore, the *b* value is the slope when turning Eq. 1 into a linear equation, which was shown in Fig. 3e (*b*_anode_ is 0.63 and *b*_cathode_ is 0.71). Admittedly, *b* value equaled to 0.5 means diffusion-controlled and 1 means capacitive contribution. To further determine the capacitive contribution ratios, Eq. 2 is analyzed:

2 $$i = k_{1} \nu + k_{2} \nu^{1/2}$$

where *k*_1_ and *k*_2_ are the constant, *k*_2_*ν*^1/2^ and *k*_1_*ν* correspond to diffusion and surface effects, respectively. From Fig. 3f, it's obvious that the capacitive contribution progressively increases with the increase in sweep rate and the contribution ratios of 3 mV s^−1^ is 88.2%. Figure S12d is the corresponding integral area diagram. It is speculated that the TiO_2_ ww/CN electrode has better electrochemical performance in LHCs from the result of high contribution ratios. It was shown in Fig. S13 the Nyquist plots of TiO_2_ ww/CN electrode and TiO_2_/CN electrode in LHCs are composed of a high-frequency semicircle, intermediate frequency semicircle, and low-frequency straight line. The plots can be fitted according to the equivalent circuit shown as the inset in Fig. S13. The *R*_s_ is ohm resistance, *R*_f_ represents the resistance of SEI film, and *R*_ct_ is the resistance of charge transfer. Warburg impedance (W1) is associated with Li^+^ diffusion in the solid phase. CPE1 is an analog constant-phase element that is related to the SEI film capacitor, and CPE2 is in connection with double-layer capacitance [49–51]. As shown in Table S1, TiO_2_ ww/CN electrode has smaller values of *R*_s_, *R*_f_, and *R*_ct_ (1.3, 93.8, and 115.5 Ω) comparing with the TiO_2_/CN electrode (1.5, 240.0, and 405.0 Ω), indicating it possesses faster kinetics in the electrochemical reaction.

To reveal the practical application feasibility of the sample, the full cells were fabricated between activated LFP cathodes and activated TiO_2_ ww/CN film anodes. The cycling performance of the LFP electrode in LHC after activating 1 cycle at 0.1 A g^−1^ (Fig. S14a) and homologous discharge/charge curves in the range of 2.5–4.3 V (Fig. S14b) manifest cyclic stability. The cycling performance of the full cell can be measured in Fig. 3g. The (−)TiO_2_ ww/CN-LFP(+) full cell exhibits high coulombic efficiency of 90% after 15 cycles of activation and then possesses coulombic efficiency with 97% over 100 cycles. Laudably, it can deliver the discharge capacity and charge capacity of 109 and 106 mAh g^−1^ at the 100th cycle, respectively. The corresponding discharge/charge curves of the full cell shown in Fig. 3h can observe the explicit charging and discharging platforms even in the 100th cycle at 0.4 A g^−1^, which indicating remarkable cycling stability. In Fig. 3i, the (−)TiO_2_ ww/CN-LFP(+) full cell can glow 22 LEDs without a hitch, demonstrating the real application.

After 1000 cycles, the TiO_2_ ww/CN electrode at 0.4 A g^−1^ was disassembled from the testing cell for TEM tests. The after-cycled electrode was completely soaked in dimethyl carbonate (DMC) for 8 h to remove the electrolyte residue on the film before testing. The shape of the after-cycled electrode is shown in the upper right corner of Fig. S15a. It can be observed from Fig. S15a that the wire-in-wire structure is well maintained. EDX elemental mapping image in Fig. S15b shows that the C, Ti, O elements are well distributed in the nanofibers [52]. It can be considered that the structure and morphology of the after-cycled electrode keep intact after long-term cycling. The lattice space observed in Fig. S15c is 3.5 Å, corresponding to the (101) plane of anatase, and the FFT further observed the (101) plane. To assess the electrochemical performance of the TiO_2_ ww/CN electrode, this work and the other partly reported binder-free and free-standing TiO_2_ anode materials in LHCs in the recent five years are compared in Table S2. Compared with rGO modified N-doped carbon foam supporting TiO_2_ [20], foam-like 3D mesopore N-doped carbon assembling TiO_2_ nanoparticles [53], thick mesoporous TiO_2_ films [54], and preferentially oriented TiO_2_ nanotubes [55], the TiO_2_ ww/CN electrode in this work has better cycling stability and higher reversible capacity. Besides, TiO_2_ ww/CN also shows better cycling stability than TiO_2_ layer on N-doped carbon foams [15], and TiO_2_/super-aligned C nanotube [56]. EOG/TiO_2_ (B) nanosheets [16], and N-doped TiO_2_/RGO hybrids [17] could endure extra-long term cycles of 10,000 cycles; however, TiO_2_ ww/CN in this work shows advantages in the respect of specific capacity.

### Electrochemical Evaluation in KHCs and Full Cells

**Fig. 4:**
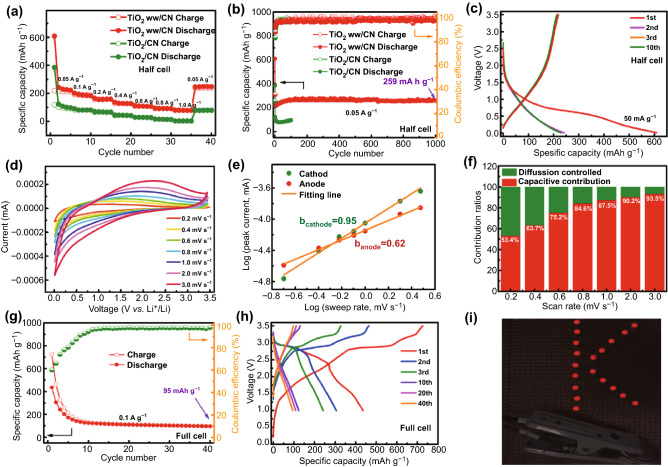
TiO_2_ ww/CN film and TiO_2_/CN film in KHCs: **a** rate performance from 0.05 to 1.0 A g^−1^, then returning back to 0.05 A g^−1^, **b** cycle performance at 0.05 A g^−1^; TiO_2_ ww/CN film in KHCs: **c** discharge–charge voltage profiles of TiO_2_ ww/CN film at 0.05 A g^−1^, **d** CV curves of TiO_2_ ww/CN film electrode from 0.2 to 3.0 mV s^−1^, **e** the log-linear relation in sweep rate ~ peak current, and **f** columnar diagram of contribution ratios from 0.2 to 3.0 mV s^−1^; (−)TiO_2_ ww/CN-PTCDA(+) full cell: **g** cycling performance, and **h** corresponding discharge–charge voltage profiles at 0.1 A g^−1^, **i** the digital picture of 19 LEDs after being lighted

It indicates the rate performance of the TiO_2_ ww/CN electrode and the TiO_2_/CN electrode in Fig. 4a. At 0.05, 0.1, 0.2, 0.4, 0.6, 0.8, and 1 A g^−1^, the reversible discharge capacities of TiO_2_ ww/CN electrode are 222, 184, 159, 127, 106, 91, and 78 mAh g^−1^, respectively. When backed to 0.05 A g^−1^ after 35 cycles, TiO_2_ ww/CN electrode recovers the discharge capacity of 248 mAh g^−1^. The rate capability of the TiO_2_ ww/CN electrode is significantly improved than the TiO_2_/CN electrode. Figure 4b shows the cycle performance of the TiO_2_ ww/CN electrode and the TiO_2_/CN electrode in KHCs at 0.05 A g^−1^. It can be observed that the TiO_2_/CN electrode has an initial discharge capacity of 285 mAh g^−1^, then it has an initial charge capacity of 87 mAh g^−1^, with ICE of 30%. TiO_2_ ww/CN electrode shows a much higher specific capacity and much better cyclability than the TiO_2_/CN electrode. It shows a discharge capacity of 243 mAh g^−1^ at the second cycle, and the discharge capacity increases gradually upon cycling. The discharge capacity is 258 mAh g^−1^ at the 60 cycles. After 1000 cycles, the discharge capacity of 259 mAh g^−1^ could be obtained with coulombic efficiencies of nearly 100%. The discharge/charge curves of the TiO_2_ ww/CN electrode at 0.05 A g^−1^ are shown in Fig. 4c. What can be observed is, the initial discharge capacity reaches 608 mAh g^−1^ and the initial charge capacity is 219 mAh g^−1^; therefore, it can be calculated that the ICE is 36%. The second, the third, and the tenth curves have unexceptionable repetition, which proves the outstanding cycling stability. The K-storage performances of the TiO_2_ ww/CN film electrode are also investigated. KHCs have the same working mechanism as LHCs and approximate standard reduction potential compared with LHCs; however, there is a huge difference in energy storage capacity between LHCs and KHCs. DFT calculations were carried out to reveal the intrinsic property of TiO_2_ when utilized as the electrode of LHCs and KHCs. The total density of states (TDOS) of dilute Li/K intercalated TiO_2_ is shown in Fig. S16a. It can be found that the alkali metal ions can generate intermediate states in the bandgap, which are slightly lower than the Fermi level. The gap between the intermediate states and conduction band is around 1.2 eV, suggesting that both the Li and K ion intercalated TiO_2_ have relatively good electronic conductivity. However, for the ionic diffusion barrier of Li/K ions in anatase TiO_2_ (1 Li/K atom intercalated in a (2 × 2 × 3) supercell), as previously revealed by us [29], the diffusion of K^+^ ions in TiO_2_ has a much larger kinetic barrier (0.70 eV) than that of Li^+^ ions (0.34 eV), which is indicative of the sluggish reaction kinetics of TiO_2_ as the electrode of KHCs. In consequence, it can conclude that the reaction of TiO_2_ in KHCs is primarily restricted by the ionic conductivity, which could be significantly improved by reducing the particle size of TiO_2_. Figure S16b shows CV curves from the first to three cycles of TiO_2_ ww/CN in 0.01–3.5 V (V vs. K^+^/K) at 0.1 mV s^−1^. The obvious cathodic peak at 0.2 V in the first scan is mainly attributed to the formation of SEI film. The 3.5–1.15 V of the cathodic process and the 2.28–3.5 V of the anodic process are the K^+^ adsorption/deadsorption, corresponding to the pseudo-capacitive effect [18]. The 1.15–0.01 V of the cathodic process and the 0.01–2.28 V of the anodic process are the K^+^ intercalation/deintercalation [18]. The area surrounded by the CV curve in the third scan is slightly larger than that in the second scan, indicating the electrochemical activation in the first few cycles. Similar to TiO_2_ ww/CN electrode in LHCs, capacitive contribution in KHCs is shown in Figs. 4d–f and S16c referencing to Eqs. 1 and 2. Under the influence of ohmic impedance and polarization, CV curves (in Fig. 4d) are gradually distorted from 0.2 mV s^−1^ to 3.0 mV s^−1^. It can be seen from Fig. 4e that b_anode_ values (0.62) in KHCs are close to *b*_anode_ values (0.63) in LHCs; however, b_cathode_ values (0.95) in KHCs is extremely higher than *b*_anode_ values (0.71) in LHCs. The contribution ratios in varying sweep speed and corresponding integral area curve in 3.0 mV s^−1^ are described in Fig. 4f and S16c, respectively. It notes that the capacitive contribution ratios of the TiO_2_ ww/CN electrode in KHCs are larger than those in LHCs. For example, the capacitive contribution ratio in KHCs is as high as 93.5% at 3.0 mV s^−1^, which is larger than that in LHCs (88.2%). Despite the large diffusion barrier of K in anatase TiO_2_ (0.70 eV) [29], TiO_2_ ww/CN film shows attractive K-storage performance, which could be in keeping with the pseudocapacitance contribution and large specific surface area.

The cycling performance at 0.1 A g^−1^ (Fig. S17a) and corresponding discharge/charge curves (Fig. S17b) in 1.5–3.5 V of PTCDA in KHC demonstrate that it has good cycle stability and obvious charging/discharging platforms. The cycling performance (Fig. 4g) and corresponding discharge/charge curves (Fig. 4h) of (−)TiO_2_ ww/CN-PTCDA(+) full cells also display high storage capacity and outstanding cycling stability. It could deliver initial charge/discharge capacities of 726/435 mAh g^−1^ with a coulombic efficiency of 60% at 0.1 A g^−1^, after 8 cycles of activation, the coulombic efficiency gradually approached 90%. At last, the charge/discharge capacities can be maintained at 95/93 mAh g^−1^ over 40 cycles with a coulombic efficiency of 97% (Fig. 4g), showing a high storage capacity. The unambiguous charging/discharging platforms even after 40 cycles in Fig. 4h further demonstrate its excellent cyclic stability. Desirably, the 100% charged (−)TiO_2_ ww/CN-PTCDA(+) full cell can allow the 19 LEDs light shine (Fig. 4i).

The TiO_2_ ww/CN electrode after 1000 cycles in KHCs at 0.05 A g^−1^ was taken apart and washed with dimethyl carbonate for TEM tests. The digital photo shown as the inset in Fig. S18a indicates that the TiO_2_ ww/CN film is intact after 1000 cycles. Figure S18a shows the wire-in-wire hierarchical structure of nanofibers remains well and the surface is slightly rough which may be caused by the formation of SEI film. The EDX elemental mapping images in Fig. S18b shows that K, C, Ti, O elements are well distributed in the fiber. As shown in the HRTEM image (Fig. S18c), the lattice fringes space and the FFT are all 3.5 Å, corresponding to the (101) crystal plane of anatase TiO_2_. The comparison results of this work with previous reported TiO_2_ anode materials in KHCs are listed in Table S3. This work shows better cycling stability than lepidocrocite-type layered TiO_2_ and MXene-derived TiO_2_/RGO [19, 20]. Hierarchical TiO_2_-C micro-tubes [18] could deliver 133 mAh g^−1^ after 1200 cycles at 0.5 A g^−1^, which showed outstanding electrochemical properties. It notes that the TiO_2_ ww/CN electrode in this work is a binder-free and free-standing electrode, demonstrating commendable mechanical flexibility.

## Conclusions

In this work, electrospun TiO_2_ ww/CN film with a unique hierarchical wire-in-wire nanostructure and excellent mechanical flexibility was fabricated. As free-standing electrodes, the film demonstrated highly improved electrochemical performance in Li/K ion batteries. It delivered discharge capacities of 497 mAh g^−1^ after 1000 cycles at 0.4 A g^−1^ and 303 mAh g^−1^ after 6000 cycles at 5 A g^−1^ in Li half-cells, and a discharge capacity of 259 mAh g^−1^ at 0.05 A g^−1^ after 1000 cycles in K half-cells. Despite the large K^+^ ions diffusion barrier in TiO_2_, the K-ion storage performance of TiO_2_ ww/CN was found to be enhanced by the high pseudocapacitance contribution (93.5% under 3.0 mV s^−1^). When TiO_2_ ww/CN film was directly used as anodes and matched with corresponding cathodes (LFP or PTCDA) to assemble the full cells, it showed high discharge capacities, excellent cyclic stability, and attractive practical application (lighting more than 19 LEDs at least). Therefore, TiO_2_ ww/CN film has broad application prospects as anode materials for LIBs and KIBs.

## Supplementary Information

Below is the link to the electronic supplementary material.

Supplementary file 1 (PDF 1516 KB)

Supplementary file 2 (MP4 2731 KB)
