# Supplementary material for: Wire-in-Wire TiO2/C Nanofibers Free-Standing Anodes for Li-Ion and K-Ion Batteries with Long Cycling Stability and High Capacity
Source: Nanomicro Lett. 2021 Apr 9;13:107. doi: 10.1007/s40820-021-00632-4 (PMC8035377; doi:10.1007/s40820-021-00632-4)
Supplement: Supplementary file 1 — (PDF 1516 KB) [file 40820_2021_632_MOESM1_ESM.pdf]

Supporting information for

Wire-In-Wire TiO_2/C Nanofibers Free-Standing Anodes for Li-Ion and K-Ion Batteries with Long Cycling Stability and High Capacity

Die Su[1], Yi Pei[2], Li Liu[1,] *, Zhixiao Liu[3,] *, Junfang Liu[1], Min Yang [1], Jiaxing Wen[1], Jing Dai[1], Huiqiu Deng[4], Guozhong Cao[2,] *

[1]National Base for International Science & Technology Cooperation, National Local Joint Engineering Laboratory for Key materials of New Energy Storage Battery, Hunan Province Key Laboratory of Electrochemical Energy Storage and Conversion, School of Chemistry, Xiangtan University, Xiangtan 411105, P. R. China

[2]Department of Materials Science and Engineering, University of Washington, Seattle, WA 98195-2120, United States

[3]College of Materials Science and Engineering, Hunan University, Changsha 410082, P. R. China

[4]School of Physics and Electronics, Hunan University, Changsha 410082, P. R. China

*Corresponding authors. E-mail: liulili1203@126.com (Li. Liu); zxliu@hnu.edu.cn (Zhixiao. Liu); gzcao@uw.edu (Guozhong. Cao)

Supplementary Tables and Figures

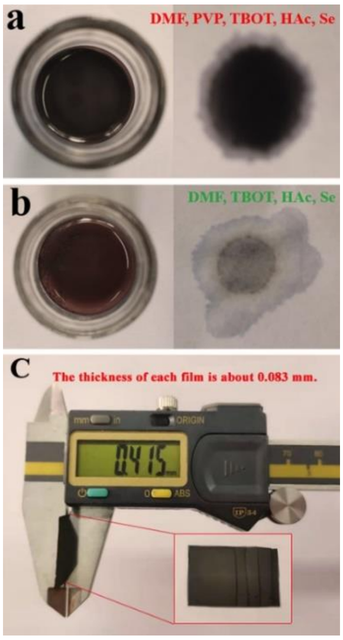

Fig. S1 Digital photos of **(a)** precursor solution (including DMF, PVP, TBOT, HAc, and Se) and **(b)** the solution without PVP, **(c)** the digital photo of the thickness of TiO_2 ww/CN film (There are five films here, thus every film is about 0.083 mm)

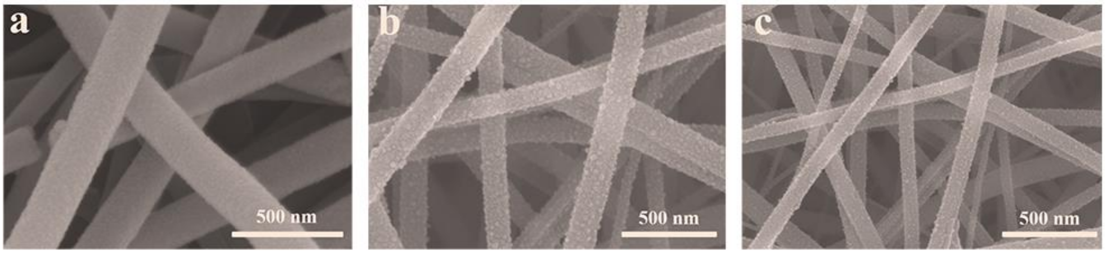

Fig. S2 FE-SEM images of **(a)** the precursor nanofibers of TiO_2 ww/CN; **(b)** the precursor nanofibers and **(c)** the carbonized nanofibers for TiO_2/CN

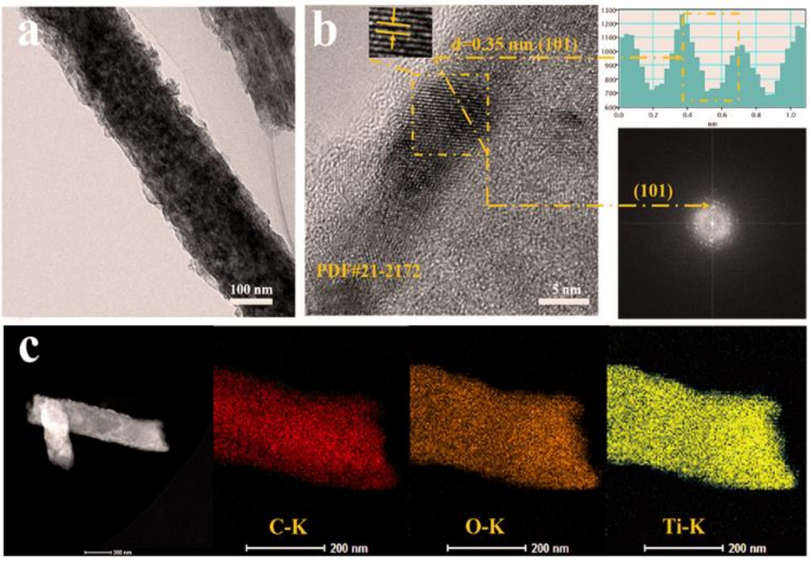

Fig. S3 FE-TEM images of TiO_2/CN: **(a)** FE-TEM image, **(b)** HRTEM images, and **(c)** EDX elemental mapping images of C, O, Ti

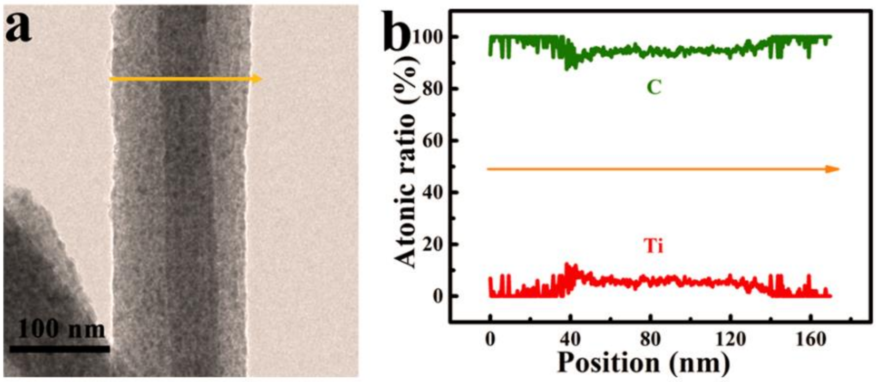

Fig. S4 (a) FE-TEM image and **(b)** the corresponding TEM-EDX lines of the atomic ratio of the elements (C and Ti) of TiO_2 ww/CN

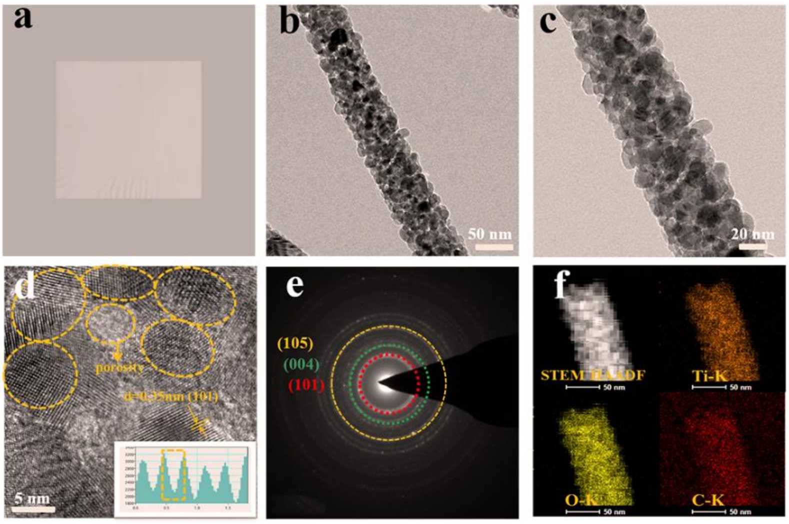

Fig. S5 (a) Digital photo, **(b, c)** FE-TEM images, **(d)** HRTEM images, **(e)** crystal diffraction ring, and **(f)** EDX elemental mapping image of TiO_2 nanowires film obtained from burning TiO_2 ww/CN film in the air at 400 °C for 2h

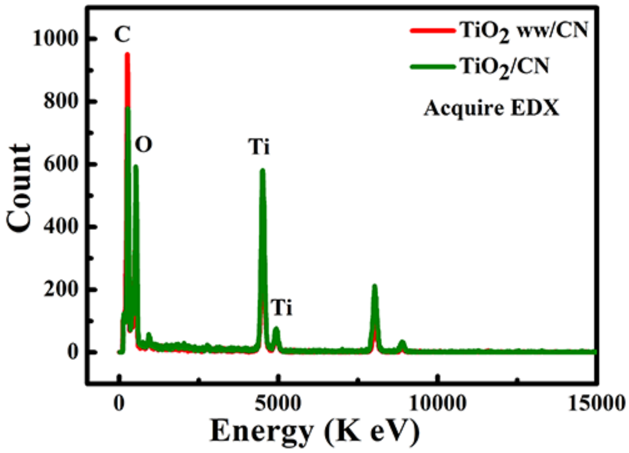

Fig. S6 EDS spectrum form FE-TEM of TiO_2 ww/CN and TiO_2/CN

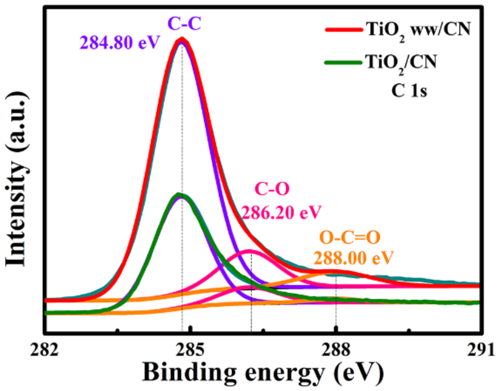

Fig. S7 High resolution C 1s of TiO_2 ww /CN and TiO_2/CN

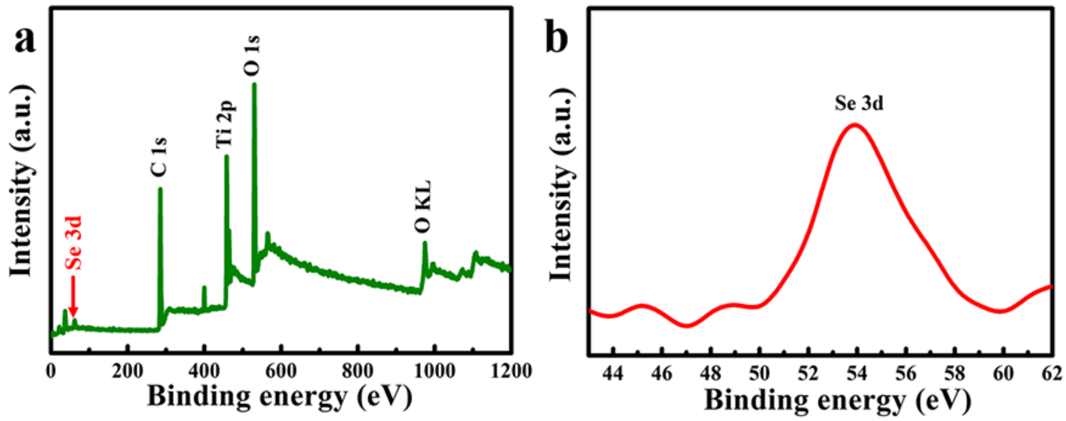

Fig. S8 (a) XPS survey spectra and **(b)** the high resolution Se 3d of the precursor solution of TiO_2 ww/CN

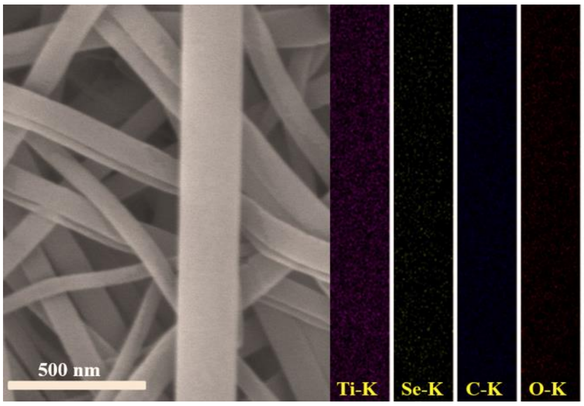

Fig. S9 FE-SEM image EDAX of the precursor nanofibers of TiO_2 ww/CN

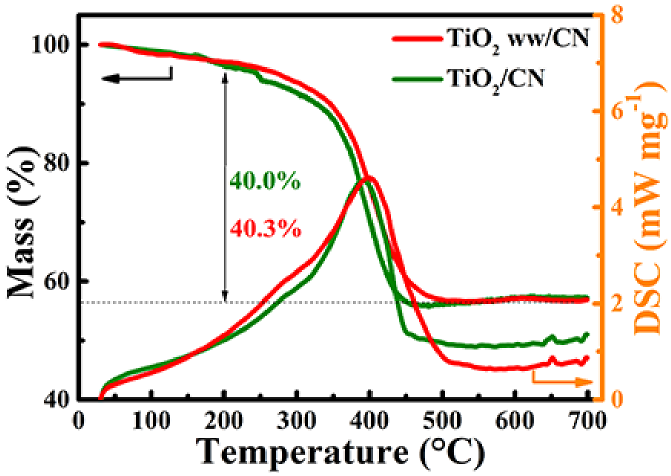

Fig. S10 TG curves in the air at 5 °C min^{-1} of TiO_2 ww/CN and TiO_2/CN

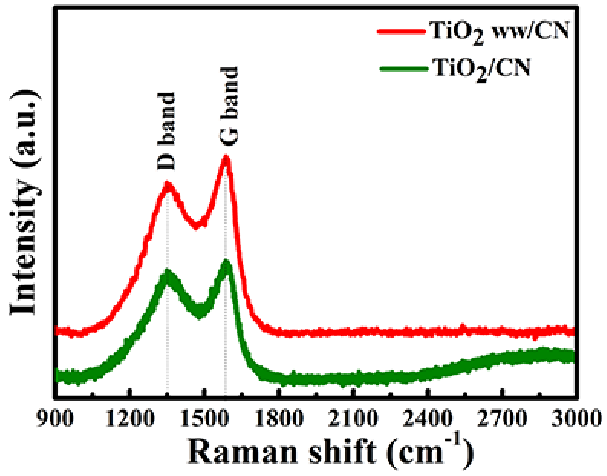

Fig. S11 Raman spectrums of TiO_2 ww/CN and TiO_2/CN

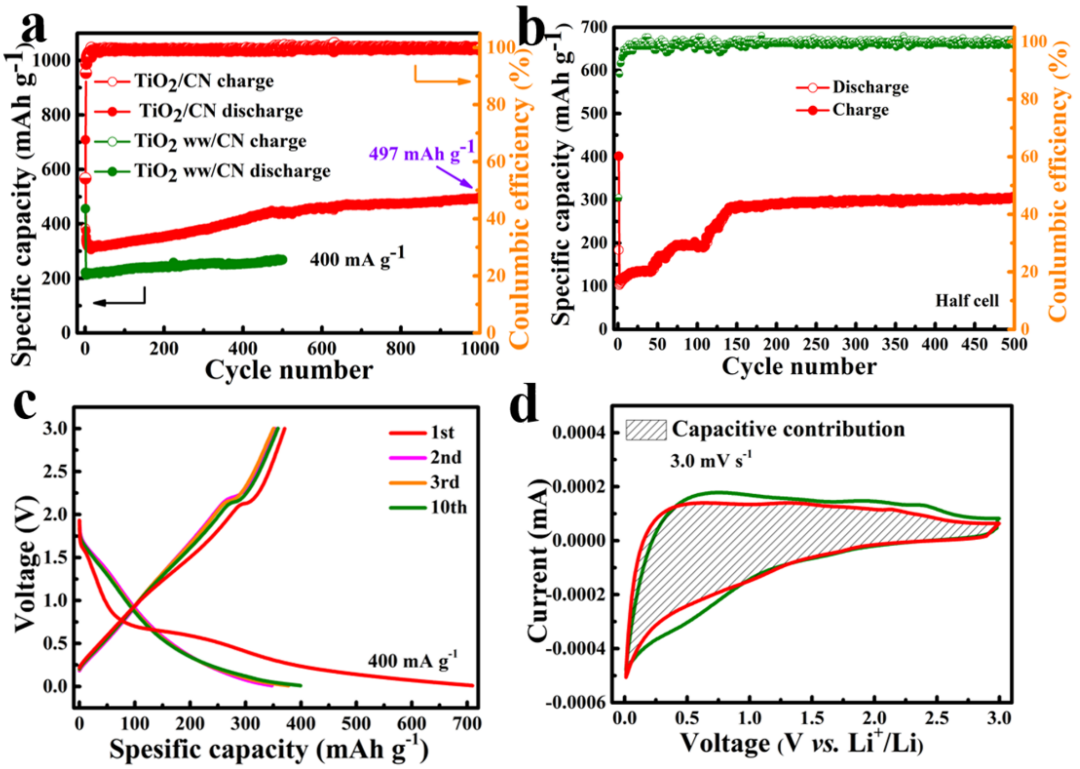

Fig. S12 TiO_2 ww/CN in LHCs: **(a)** cycling stability of TiO_2 ww/CN electrode and TiO_2/CN electrode, **(b)** cycling performance at 5 A g^{-1} for TiO_2 ww/CN electrode at first 500 cycles, **(c)** discharge-charge voltage profiles of TiO_2 ww/CN electrode at 400 mA g^{-1}, and **(d)** the integral area diagram of TiO_2 ww/CN film electrode in 3.0 mV s^{-1}

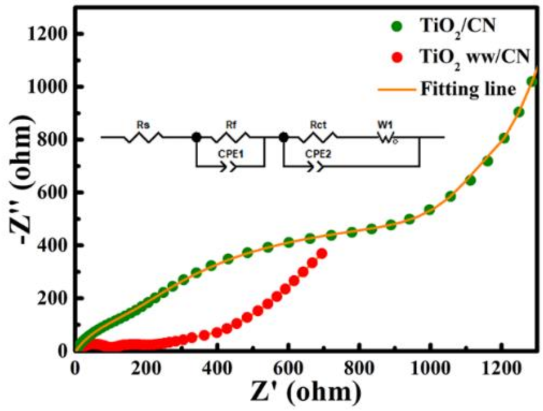

Fig. S13 Nyquist plots of TiO_2/CN and TiO_2 ww/CN after 1 cycle at a current density of 50 mA g^{-1} in LHCs, the insert section is the equivalent circuit

Table S1 Simulated impendence parameters (R_s, R_f,, and R_{ct}) of both TiO_2 ww/CN and TiO_2/CN in LHCs

Samples	R_s (Ω)	R_f (Ω)	R_{ct} (Ω)
TiO_2 ww/CN	1.3	93.8	115.5
TiO_2/CN	1.5	240.0	405.0

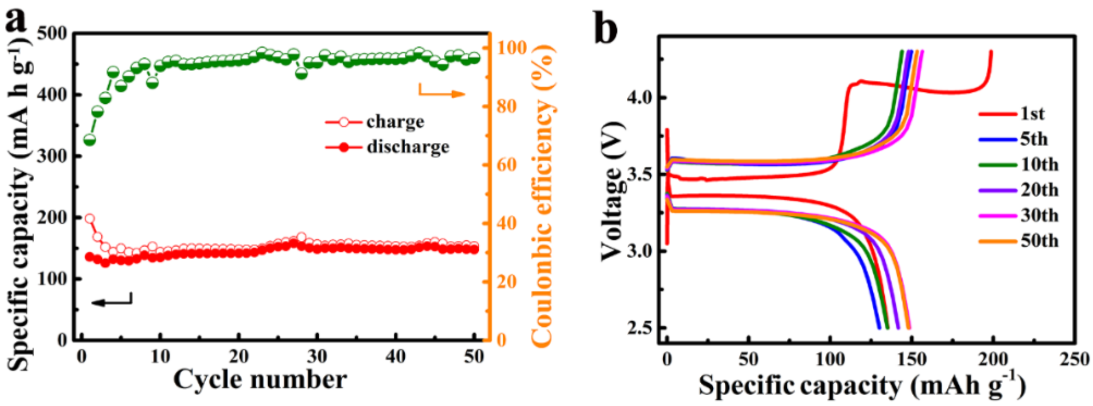

Fig. S14 LFP electrode in LHC: (a) cycling performance at 0.4 A g^{-1} after activating 1 cycle at 0.1 A g^{-1} and **(b)** discharge-charge voltage profiles in the range of 2.5-4.3 V

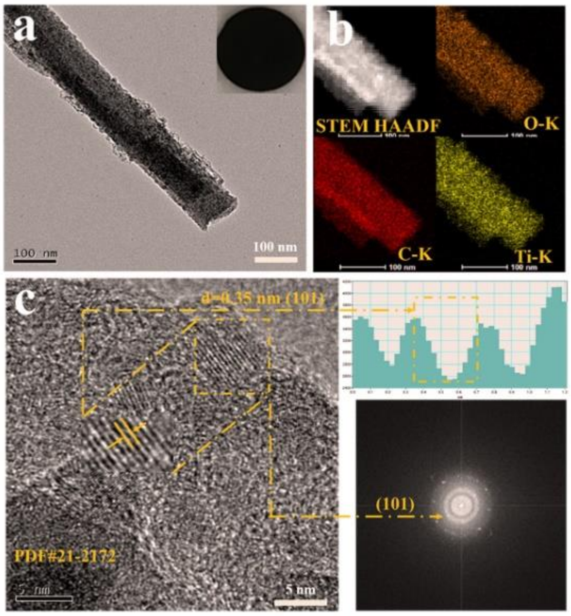

Fig. S15 **(a)** TEM image and the shape of after-cycled electrodes (insert section), **(b)** EDX elemental mapping images, and **(c)** HRTEM images for the TiO_2 ww/CN electrode after 1000 cycles in LHC

Table S2 Electrochemical performance comparison of TiO_2 ww/CN electrode with other partly reported binder-free and free standing TiO_2 anode materials in LHCs in recent five years

Samples	Synthetic method	Current density (mA g^{-1})	Capacity	Voltage range (V)	Refs.
rGO modified N-doped carbon foam supporting TiO_2	rGO to wrap around the 3D NCF by hydrothermalpyrolysis	335	214 mAh g-1 after 150 cycles	1.0-3.0	10.1016/j.electacta.2019.04.136
Foam-like, 3D mesopore N-doped carbon assembling TiO_2 nanoparticles	boiling bubbles as template	168	223 mAh g^{-1} after 200 cycles	0.01-3.0	10.1016/j.jpowsour.2019.02.094
Thick mesoporous TiO_2 films	sol–gel templating using pluronic F127 surfactant	~110	254 mAh g^{-1} after 200 cycles	1.0-3.0	10.1002/adfm.201801849
Preferentially oriented TiO_2 nanotubes	two-step anodization process	16.8	145 mAh g^{-1} after 330 cycles	1.1-3.0	10.1021/acsami.7b11388

TiO_2 layer on N-doped carbon foams	foams as template, then hydrolysis	1000	149 mAh g-1 after 100 cycles	1.0-3.0	10.1002/smll.201602179
TiO_2/super-aligned C nanotube	SACNT array with TBOT via sol-gel	10110	~100 mAh g^{-1} after 1000 cycles	1.0-3.0	10.1021/acssuschemeng.7b03671
EOG/TiO_2(B) nanosheets	EOG foam loaded with oriented TiO_2 (B) nanosheets	500	~325.5 mAh g^{-1} after 10000 cycles	1.0-3.0	10.1016/j.nanoen.2016.01.010
N-doped TiO_2/RGO hybrids	ultrasonication	1675	182.7 mAh g^{-1} after 10000 cycles	1.0-3.0	10.1021/acsaem.8b00836
**TiO_2 ww/CN**	**electrospun**	**400 and 5000**	**497 mAh g^{-1} after 1000 cycles and 303 mAh g^{-1} after 6000 cycles**	**0.01-3.0**	**This work**

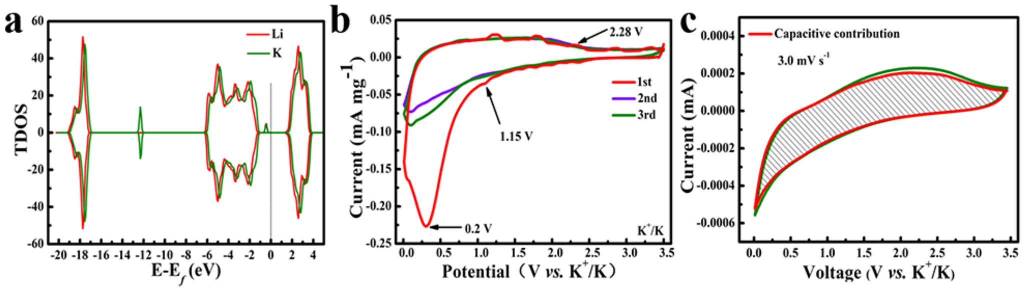

Fig. S16 The TiO_2 ww/CN electrode and TiO_2/CN electrode of **(a)** TDOS of dilute Li and K in TiO_2, **(b)** CV curves of TiO_2 ww/CN film in KHC at 0.1 mV s^{-1}, and **(c)** the integral area diagram of TiO_2 ww/CN film electrode in KHC at 3.0 mV s^{-1}

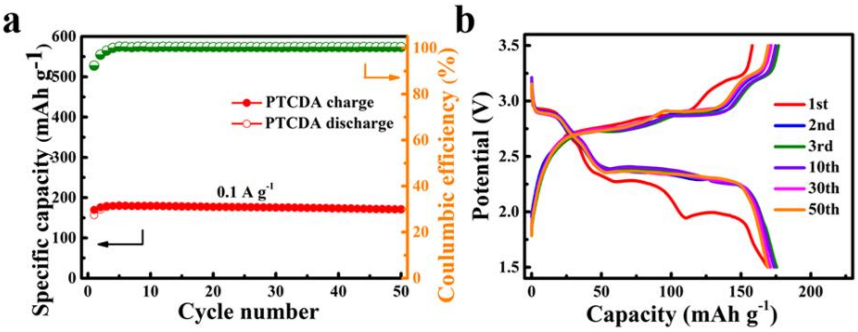

Fig. S17 PTCDA electrode in KHCs: (a) cycling performance at 0.1 A g^{-1} and **(b)** discharge-charge voltage profiles in the range of 1.5-3.5 V

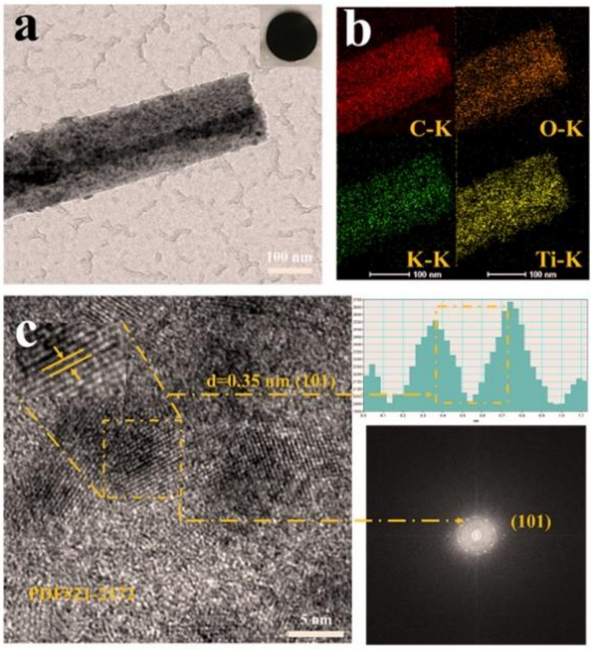

Fig. S18 **(a)** FE-TEM image and the shape of the after-cycled electrode (upper light), **(b)** EDX elemental mapping image, and **(c)** HRTEM images for TiO_2 ww/CN after 1000 cycles in KHC

Table S3 Electrochemical performance comparison of the TiO_2 ww/CN electrode with other reported TiO_2 anode materials in KHCs

Samples	A: B: C * (wt. %)	Current density (mA g^{-1})	Capacity	Voltage range (V)	Reference
Lepidocrocite-Type Layered TiO_2	80: 10: 10	25	37 after 45 cycles	0.01-2.0	10.1021/acsaem.8b00170
MXene-derived TiO_2/RGO	80: 10: 10	200	155 mAh g^{-1} after 200 cycles	0.01-3.0	10.1039/C8TA12069B
Hierarchical TiO_2-C micro-tubes	super P and CMC in water	500	133 mAh g^{-1} after 1200 cycles	0.01-3.0	10.1016/j.nanoen.2019.03.002
**TiO_2 ww/CN**	**Binder free and free standing**	**50**	**259 mAh g^{-1} after 1000 cycles**	0.01-3.5	**This work**

* A: B: C = active materials: binder: conductive additive
